# Using special Janus nanobelt as constitutional unit to construct anisotropic conductive array membrane for concurrently affording color-tunable luminescence and superparamagnetism†

**DOI:** 10.1039/c8ra06283h

**Published:** 2018-09-10

**Authors:** Xue Xi, Wensheng Yu, Qianli Ma, Dan Li, Xiangting Dong, Jinxian Wang, Guixia Liu

**Affiliations:** Key Laboratory of Applied Chemistry and Nanotechnology at Universities of Jilin Province, Changchun University of Science and Technology Changchun 130022 China maqianlimail@cust.edu.cn +86-0431-85383815; Changchun University of Science and Technology China xtdong@cust.edu.cn +86-0431-85582574

## Abstract

We have used electrospinning technology to fabricate a tri-functional nanobelt array membrane exhibiting tunable anisotropic electrical conduction, superparamagnetism and color-tunable luminescence by using a lab-made co-axis//single-axis spinneret and an aluminum drum collection device. Each one-dimensional (1D) Janus nanobelt is composed of luminescent-superparamagnetic bifunctional [Fe_3_O_4_/polymethyl methacrylate (PMMA)]@[Tb(BA)_3_phen/Eu(BA)_3_phen/PMMA] coaxial nanobelt and conducting polyaniline (PANI)/PMMA nanobelt. Moreover, all Janus nanobelts are aligned in the same manner to generate a two-dimensional (2D) array film. The conductance along the length is much stronger than the conductance in the width (two perpendicular directions). Therefore, the array membrane has excellent anisotropic electrical conduction. The conduction ratio reaches 10^8^ times between the length and width of the Janus nanobelt array membrane, which is the highest conduction ratio between the two perpendicular directions for nanobelt materials reported internationally. Furthermore, we can modulate the degree of electrically conducting anisotropy of the samples by varying the amount of PANI. In addition, the Janus nanobelt array membrane is concurrently endowed by superior and adjustable superparamagnetism and photoluminescence. Importantly, the innovative philosophy and manufacturing technique of the new Janus nanobelt array membrane provide an easy way to prepare multifunctional nano-membranes.

## Abstract 2

Flexible peculiar-structured Janus nanobelts and their array membranes of high electrically conductive anisotropy, luminescence and superparamagnetism were fabricated *via* electrospinning.

## Graphical abstract

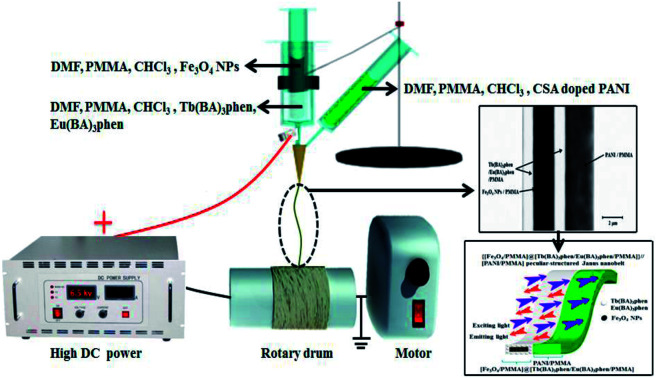

## Introduction

Anisotropic conducting film (ACF) is a new type of electronic composite material, which exhibits one direction conduction and other direction insulation. ACFs are widely used in micro-electronic packages, electrode conjuncture and other fields. ACFs can be divided into type I and type II ACFs in accordance with their conducting properties. In terms of type I ACFs, the directions along the film thickness and surface are conducting and insulating, respectively. The preparation technology of type I ACFs is advanced and widely used in the manufacture of modern electronic devices. Type II ACFs are single-layer or multi-layer thin films composed of parallel 1D conducting cells with different conductivities and different directions on the surface. These 1D conductors are mainly ultrafine fibers or long chains of molecules that guide electricity, which are separated by weak conductors such as air and/or interfaces. According to this particular structure, electrons move along the length of a 1D conducting element, but they are blocked vertically by air or an interface. Therefore, high conductivity can be observed along the length of the conducting element; however, low conductivity can be observed perpendicular to the length. Kovtyukhova *et al.*^1^ fabricated multiple layers of SWNTox/polymer membranes by conventional layer and layer assembly; the vertical conductance was 10^3^ times lower than that along the length. Liu *et al.*^2^ prepared anisotropic conducting polymer films by electrospinning. The conductivity of the film along the vertical dimension was 10^6^ times lower than that along the length. Huang *et al.*^3^ fabricated an anisotropic conducting polymer membrane. The results showed that the resistivity along the stripe was 6 orders of magnitudes higher compared with the resistivity along the orthogonal MWCNT stripe. At present, many serious weaknesses of the type II ACFs remain; thus, type II ACFs cannot be used in industry and can only be studied in the laboratory. Much research needs to be done on type II ACFs before their large-scale industrialization or applications. Therefore, solving or alleviating these defects is an urgent research topic.

There are three ways to fabricate anisotropic conducting polymer nanomaterials. The first way is to orient the conducting material into the polymer and endow the polymer film with conducting anisotropy characteristics.^4^ The second way is to apply an anisotropic conducting belt as a synthetic unit;^5,6^ for example, Ma *et al.* used the electrospinning technique to prepare an array film made of conducting anisotropic Janus nanobelts and microbelts. Because of this unique structure, the conductivities along the perpendicular and length dimensions of the belts are up to 10^8^ times in disparity. The third way is to assemble the layered synthetic material into a strip 1D chain in a polymer. On this basis, several kinds of polymers in the strip chain and one dimensional chain technology, including shear assembly within magnetic field-induced self-assembly, guided by template assembly and nano filler and block copolymer collaborative assembly can be developed.

Electrospinning is the most convenient method to convert a viscous solution into a successive fiber with diameter ranging from micron to nanometer under high voltage.^7–9^ This technology has been popularized in the sectors of regenerative biology, electrochemical materials, optical fiber humidity sensors and filtration materials. At present, multifunctional nanomaterials are one of the hot topics in research.^10^ For photoelectromagnetic nanomaterials, a previous study by our research team and other researchers demonstrated that dark colored magnetic Fe_3_O_4_ nanoparticles (NPs) and conducting PANI greatly decrease the photoluminescence properties of rare earth (RE) compounds as they are blended.^11^ Up to now, our scientific team has fabricated some multifunctional nanomaterials through electrospinning technology, which contain coaxial nanocables,^12^ coaxial nanobelts,^13^ Janus nanoribbons,^14^ and Janus nanofibers.^15,16^ The 1D structures mentioned above can offer two independent partitions: RE compounds in one partition and Fe_3_O_4_ NPs and PANI in other partition. This special nanostructure can effectively separate RE compounds from Fe_3_O_4_ NPs and PANI, resulting in significant reduction in the luminescence of RE compounds by Fe_3_O_4_ NPs and PANI. The experiment also showed that while Fe_3_O_4_ NPs and PANI are mixed in one partition, Fe_3_O_4_ NPs cause PANI to be discontinuous in the nanofiber/nanobelt, thereby reducing the electrical conductivity of the nanomaterials. It is therefore necessary to further isolate the luminescent, magnetic and electrically conducting materials if the powerful photoluminescence performance and high conductance of the multifunctional nanomaterials are to be attained. To achieve this innovative academic concept, in this study, we developed a {[Fe_3_O_4_/PMMA]@[Tb(BA)_3_phen/Eu(BA)_3_phen/PMMA]}//[PANI/PMMA] (defined as [Magnetic@Luminescent]//Electrical, abbreviated as [M@Lum]//E) Janus nanobelt to implement three separate functional partitions included in one Janus nanobelt. The [M@Lum]//E Janus nanobelt was composed of two distinct sides comprising a [Fe_3_O_4_/PMMA]@[Tb(BA)_3_phen/Eu(BA)_3_phen/PMMA] coaxial nanobelt (magnetic-luminescent side) and a PANI/PMMA nanobelt (conducting side), and the Janus nanobelt successfully completed a variety of versatile asymmetric integrations. Furthermore, by using {[Fe_3_O_4_/PMMA]@[Tb(BA)_3_phen/Eu(BA)_3_phen/PMMA]}//[PANI/PMMA] as constitutional and conducting units, a {[Fe_3_O_4_/PMMA]@[Tb(BA)_3_phen/Eu(BA)_3_phen/PMMA]}//[PANI/PMMA] Janus nanobelt array membrane (abbreviated as [M@Lum]//E JAM) was obtained *via* the parallel electrospinning technique. The array membrane combines high anisotropic electrical conduction, superparamagnetism and luminescence properties. To date, there has not been a report on such nanostructures. Moreover, to highlight the strength of [M@Lum]//E JAM, we also prepared samples of five different structures for comparison. The morphologies, structures, luminescence, superparamagnetism and electrically conducting anisotropy of the prepared samples were discussed, and we obtained some new conclusions.

## Experimental sections

### Chemicals

The chemicals are summarized in the ESI.†

### Preparation of PMMA, Fe_3_O_4_ NPs, Tb(BA)_3_phen and Eu(BA)_3_phen compounds

The preparation procedures of PMMA, Fe_3_O_4_ NPs, Tb(BA)_3_phen and Eu(BA)_3_phen compounds are given in the ESI.†

### Preparation of spinning solutions

Three types of spinning solutions were used to synthesize [M@Lum]//E JAM, and their compositions are summarized in the ESI (Tables S1–S3).† To show the superior advantages of [M@Lum]//E JAM, five contrastive membranes were prepared including {[Fe_3_O_4_/PMMA]@[Tb(BA)_3_phen/Eu(BA)_3_phen/PMMA]}//[PANI/PMMA] Janus nanobelt non-array membrane (abbreviated as [M@Lum]//E JNM), [Fe_3_O_4_/Tb(BA)_3_phen/Eu(BA)_3_phen/PMMA]//[PANI/PMMA] Janus nanobelt array membrane (abbreviated as [M-Lum]//E JAM), [Fe_3_O_4_/Tb(BA)_3_phen/Eu(BA)_3_phen/PMMA]//[PANI/PMMA] Janus nanobelt non-array membrane (abbreviated as [M-Lum]//E JNM), Fe_3_O_4_/Tb(BA)_3_phen/Eu(BA)_3_phen/PANI/PMMA composite nanobelt array membrane (abbreviated as M-Lum-E CAM), and Fe_3_O_4_/Tb(BA)_3_phen/Eu(BA)_3_phen/PANI/PMMA composite nanobelt non-array membrane (abbreviated as M-Lum-E CNM). Among them, the constructive principles of the two contrastive samples M-Lum-E CAM and CNM are obtained from the ref. 11 and 17. The spinning solutions used to prepare the contrastive samples and the components of the contrastive samples are summarized in the ESI† (Table S4†).

### Electrospinning process

**Fig. 1:**
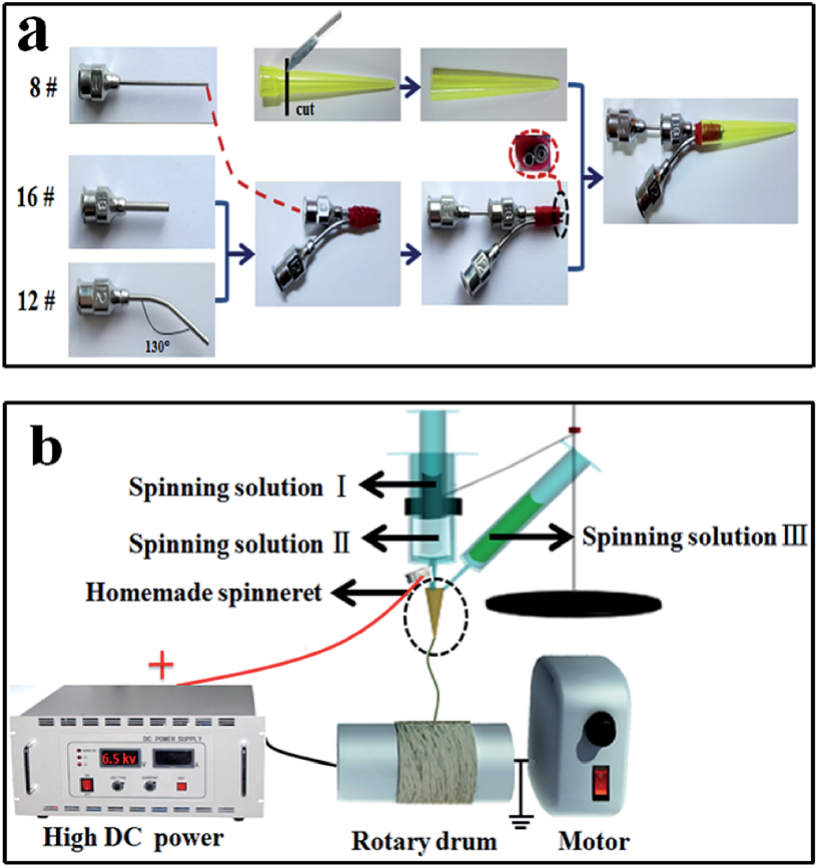
Sketch maps of the preparation procedure for specially designed and installed co-axis//single-axis spinneret (a) and electrospinning equipment for fabricating [M@Lum]//E JAM (b).

In the previous study, parallel spinnerets were used to fabricate Janus nanobelts with two isolated functional domains. The parallel spinnerets consisted of two bent stainless steel needles.^5,14^ In this study, we first designed and manufactured a new type of spinneret of co-axis//single-axis spinneret. Afterwards, novel and brand-new flexible peculiar-structured [coaxial nanobelt]//[nanobelt] Janus nanobelts with three isolated functional domains were prepared *via* electrospinning technology using the co-axis//single-axis spinneret. Thus, the co-axis//single-axis spinneret is more superior for the fabrication of nanobelts with more isolated functional domains. As shown in Fig. 1a, the specially made co-axis//single-axis spinneret consists of three different truncated stainless steel needles (16#, 12# and 8#). The 16# stainless steel needle and 8# stainless steel needle formed a coaxial spinneret, and a blended 12# stainless steel needle was strapped to the coaxial spinneret abreast with a nylon string. A plastic jet nozzle was then placed on the top of the needles. The electrospinning apparatus for preparing [M@Lum]//E JAM is depicted in Fig. 1b. The spinning solutions I–III were separately poured into the three plastic syringes. To obtain an ordered array membrane, the experimental apparatus used an aluminum revolving drum as the nanobelt receiving device, and the rotational speed was moderated to 1500 rpm. The electrospinning conditions and electrospinning equipment for fabricating contrastive samples are listed in the ESI (please see Table S4† for more details).

## Results and discussion

### Phase

**Fig. 2:**
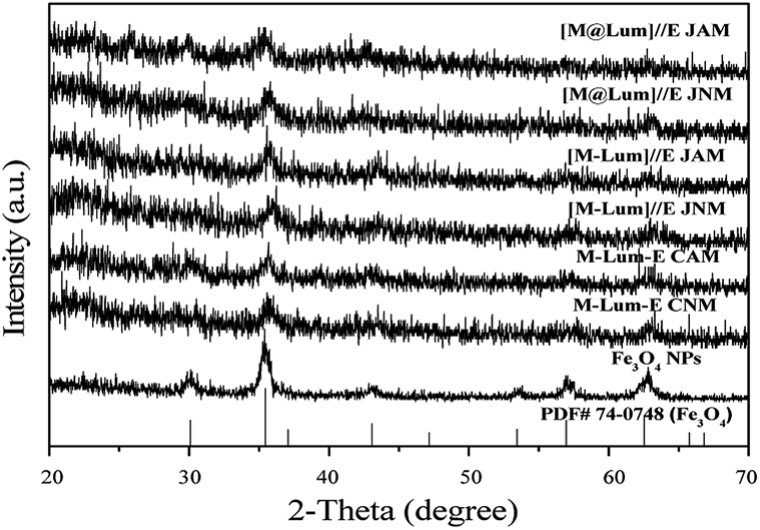
XRD patterns of the samples with PDF standard card of Fe_3_O_4_.

We characterized [M@Lum]//E JAM, five contrastive membranes and Fe_3_O_4_ NPs by X-ray diffractometry (Fig. 2). The XRD patterns of Fe_3_O_4_ NPs were in agreement with that of the cubic-structured of Fe_3_O_4_ (PDF 74-0748). The tests showed that all [M@Lum]//E JAM and contrastive samples contain Fe_3_O_4_ NPs.

### Morphology and internal structures

**Fig. 3:**
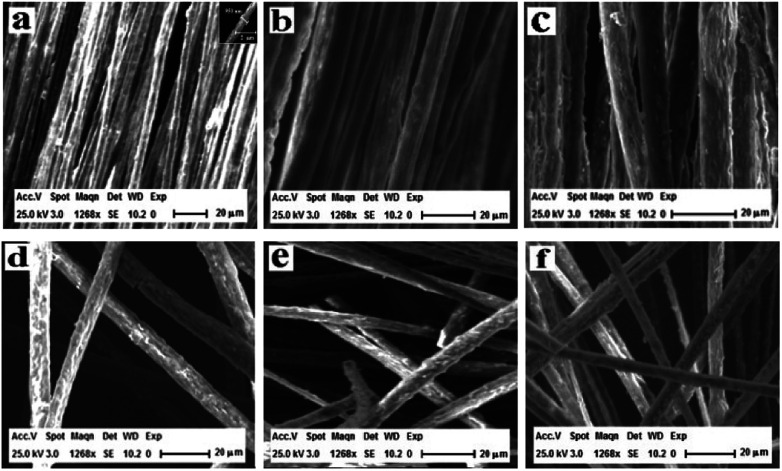
SEM images of [M@Lum]//E JAM (a), [M-Lum]//E JAM (b), M-Lum-E CAM (c), [M@Lum]//E JNM (d), [M-Lum]//E JNM (e) and M-Lum-E CNM (f).

**Fig. 4:**
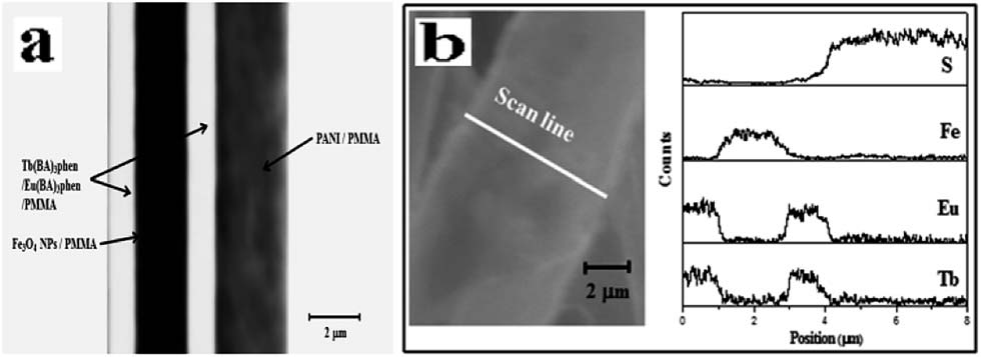
BM image (a) and EDS line-scan analysis (b) of a single [M@Lum]//E Janus nanobelt.

The scanning electron microscopy (SEM) images of [M@Lum]//E JAM are shown in Fig. 3a, which shows that nearly all the Janus nanobelts are configured towards the same dimension. The width of the [M@Lum]//E Janus nanobelts is *ca.* 6.5 μm, and the thickness is shorter than 1 μm; the results show that nearly all of the broadsides of these nanobelts are face up, which may be because this state helps the nanobelts to deposit onto the rotary drum in a much more steady manner when they are in a lower position. On the basis of the biological microscopy (BM) image analysis, the internal structure of [M@Lum]//E Janus nanobelt can be observed. A clear Janus nanobelt structure can be observed in Fig. 4a, and one side of the Janus nanobelt is [Fe_3_O_4_/PMMA]@[Tb(BA)_3_phen/Eu(BA)_3_phen/PMMA] coaxial nanobelt, which consists of a low-contrast shell and a dark-colored core; in contrast, the other side is PANI/PMMA. For further demonstration of the distinct Janus-type structure of the [M@Lum]//E Janus nanobelts, energy dispersive spectroscopy (EDS) line scan analysis was conducted (Fig. 4b); Tb, Eu, Fe and S represent Tb(BA)_3_phen, Eu(BA)_3_phen, Fe_3_O_4_ and PANI, respectively. Elemental Fe exists in the middle domain of the [Fe_3_O_4_/PMMA]@[Tb(BA)_3_phen/Eu(BA)_3_phen/PMMA] coaxial nanobelt. In addition, we found that only elemental Tb and Eu are dispersed on both sides of the coaxial nanobelt, and the other half side of the [M@Lum]//E Janus nanobelt is PANI/PMMA. Through SEM, BM, EDS line-scan analyses, we inferred that [M@Lum]//E JAM has been successfully prepared.

**Fig. 5:**
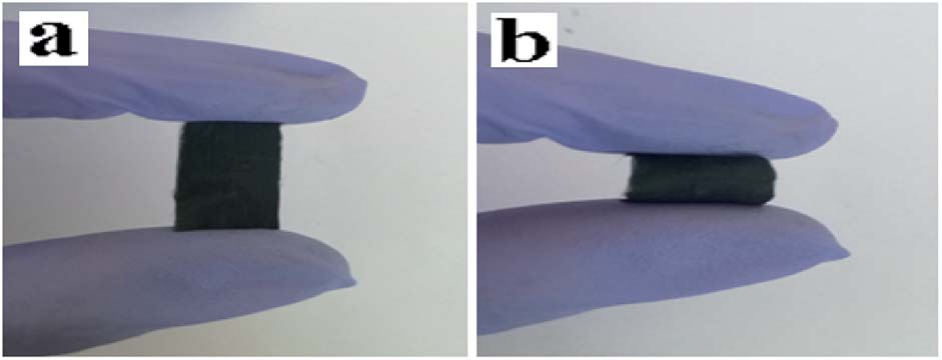
Digital photos of [M@Lum]//E JAM: (a) an unbent and (b) bent Janus nanobelt array membrane.

Fig. 3b and c show SEM images of [M-Lum]//E JAM and M-Lum-E CAM, respectively. As can be seen in Fig. 3b and c, the widths of each single [M-Lum]//E Janus nanobelt and M-Lum-E composite nanobelt are *ca.* 7.5 μm and 8 μm, respectively. In a further survey, [M-Lum]//E Janus nanobelts and M-Lum-E composite nanobelts are ordered in all samples. The SEM images of [M@Lum]//E Janus nanobelt non-array, [M-Lum]//E Janus nanobelt non-array and M-Lum-E composite nanobelt non-array are shown in Fig. 3d, e and f, respectively, whereas the Janus nanobelts and composite nanobelts are out-of-order. The widths of the independent nanobelt in [M@Lum]//E Janus nanobelt non-array, [M-Lum]//E Janus nanobelt non-array and M-Lum-E composite nanobelt non-array are *ca.* 8 μm, 7 μm and 5.5 μm, respectively. Fig. 5 shows typical digital photos of [M@Lum]//E JAM, and we can observe from the photos that the membrane can be easily bent and folded. This is very important for future applications of materials in flexible devices.

### Fluorescent property

**Fig. 6:**
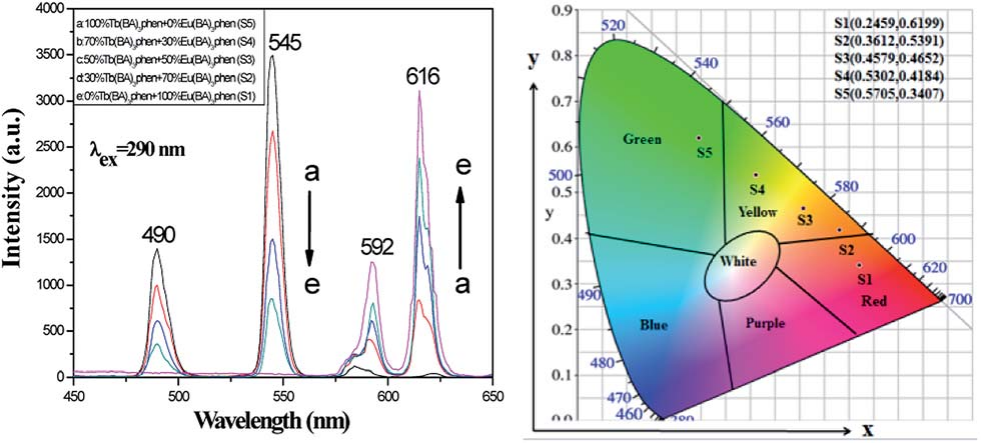
EM spectra (left) and CIE chromaticity diagram (right) of [M@Lum]//E JAM containing various mass ratios of Eu(BA)_3_phen to Tb(BA)_3_phen.

Luminescence performances of [M@Lum]//E JAM were studied. Color-tunable luminescence was obtained by modifying the ratios of Eu(BA)_3_phen to Tb(BA)_3_phen (samples [S_a2_@S_b*x*_]//S_c2_, *x* = 1–5) when the mass percentage of PANI to PMMA was fixed at 30% and the mass ratio of Fe_3_O_4_ to PMMA was fixed at 1 : 1. Based on previous experimental investigations,^18^ we chose 290 nm ultraviolet light as the excitation (abbreviated as EX) source for Tb(BA)_3_phen and Eu(BA)_3_phen compounds; under this ultraviolet light, the emission (abbreviated as EM) spectra of [M@Lum]//E JAM exhibited peaks at 490 and 545 nm, which were ascribed to the ^5^D_4_ → ^7^F_*J*_ (*J* = 6, 5) energy level transitions of Tb^3+^, and the peaks at 592 and 616 nm were due to the ^5^D_0_ → ^7^F_*J*_ (*J* = 1, 2) energy level transitions of Eu^3+^, as seen in Fig. 6 (left). The test results showed that as the mass ratio of Eu(BA)_3_phen to Tb(BA)_3_phen decreased, the green fluorescence EM of Tb^3+^ ions at 490 and 545 nm gradually increased, and the orange and red fluorescence EM at 592 and 615 nm of Eu^3+^ ions slowly decreased. Fig. 6 shows the CIE coordinates for [M@Lum]//E JAM excited by 290 nm, and the experiment verified that after adjusting the mass ratios of Eu(BA)_3_phen compounds to Tb(BA)_3_phen compounds, the fluorescence color of the samples can be adjusted from red to green.

**Fig. 7:**
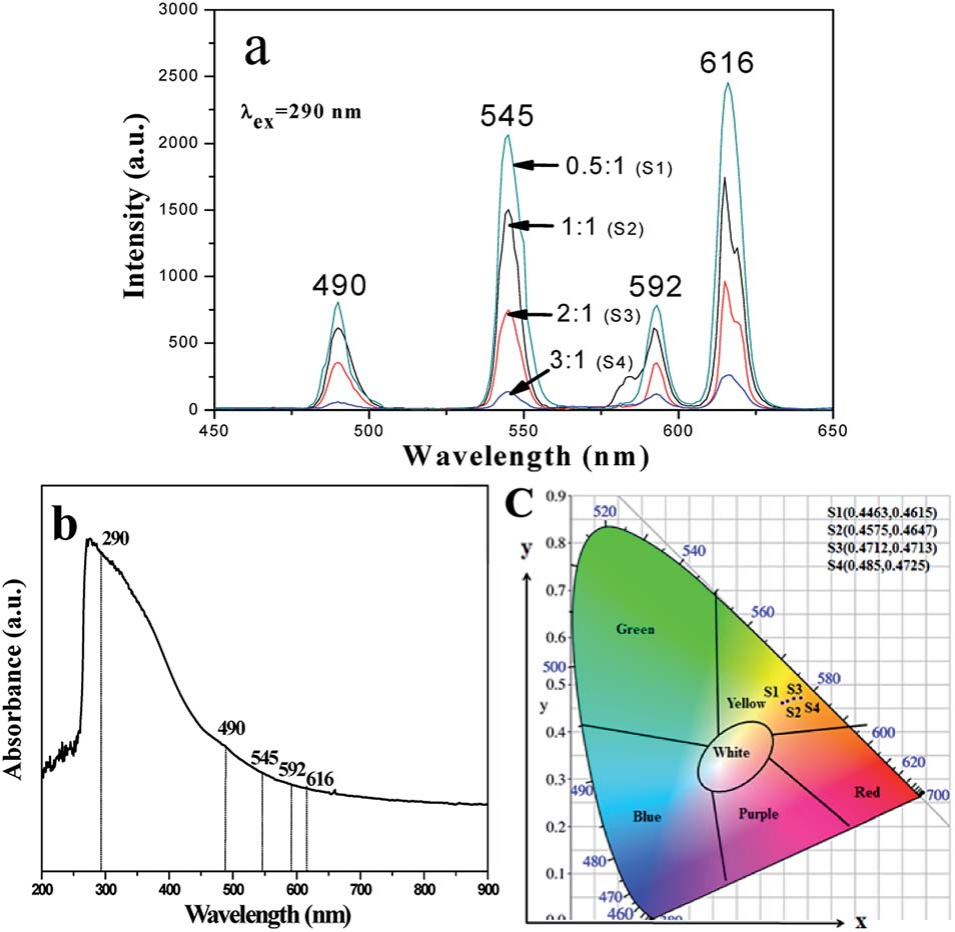
EM spectra (a) and CIE chromaticity diagram (c) of [M@Lum]//E JAM containing various mass ratios of Fe_3_O_4_ NPs to PMMA; UV-vis absorption spectrum of Fe_3_O_4_ NPs (b).

**Fig. 8:**
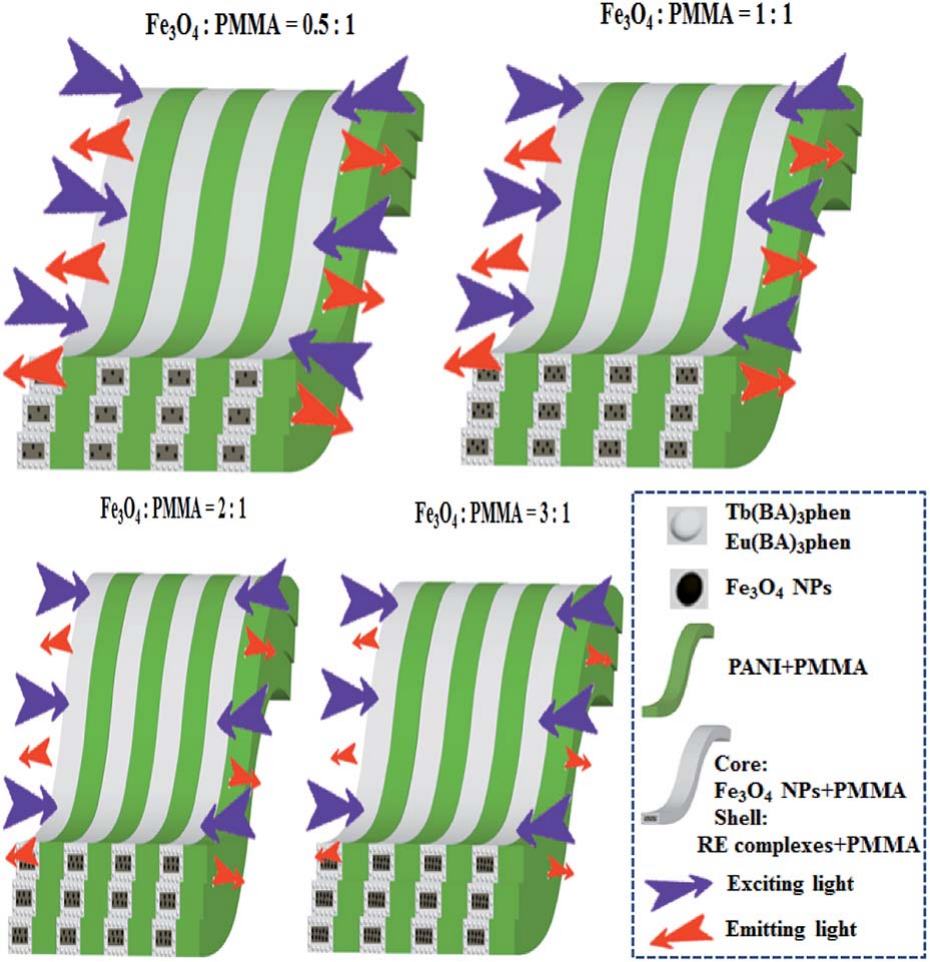
Sketch maps of EX and EM light in [M@Lum]//E JAM containing various mass ratios of Fe_3_O_4_ NPs to PMMA.

In addition, [M@Lum]//E JAM containing various amounts of Fe_3_O_4_ NPs was fabricated to detect the influence of the addition of various amounts of Fe_3_O_4_ NPs (samples [S_a*x*_@S_b3_]//S_c2_, *x* = 1–4) on the fluorescence performances of [M@Lum]//E JAM when the mass ratio of Eu(BA)_3_phen to Tb(BA)_3_phen was set at 1 : 1 and the mass percentage of PANI to PMMA was fixed at 30%, as shown in Fig. 7a. It was discovered that as the content of Fe_3_O_4_ NPs increased, the EM intensities of [M@Lum]//E JAM decreased. The results demonstrated the light absorption of Fe_3_O_4_ NPs. To explain the effect of Fe_3_O_4_ NP content on the fluorescence intensity, sketch maps of EX and EM lights in [M@Lum]//E JAM containing various mass ratios of Fe_3_O_4_ NPs to PMMA are shown in Fig. 8. Fig. 7b shows the absorption spectrum of Fe_3_O_4_ NPs. It was observed that Fe_3_O_4_ NPs can absorb visible light from 200 nm to 900 nm. Thus, the intensities of the EX and EM peaks decreased as the EX and EM lights were absorbed and weakened by Fe_3_O_4_ NPs. Fig. 7c demonstrates the CIE chromaticity diagram of [M@Lum]//E JAM with various Fe_3_O_4_ amounts under EX of 290 nm ultraviolet light. The results showed that the EM color of [M@Lum]//E JAM shifted to red with the addition of more Fe_3_O_4_ NPs, and this elaborated the theory that Fe_3_O_4_ has powerful light absorption to the light at lower wavelengths. With an increase in the amount of Fe_3_O_4_ NPs, the EM color of [M@Lum]//E JAM shifted toward red. This disclosed the fact that Fe_3_O_4_ has more powerful light absorption for light of lower wavelengths.

**Fig. 9:**
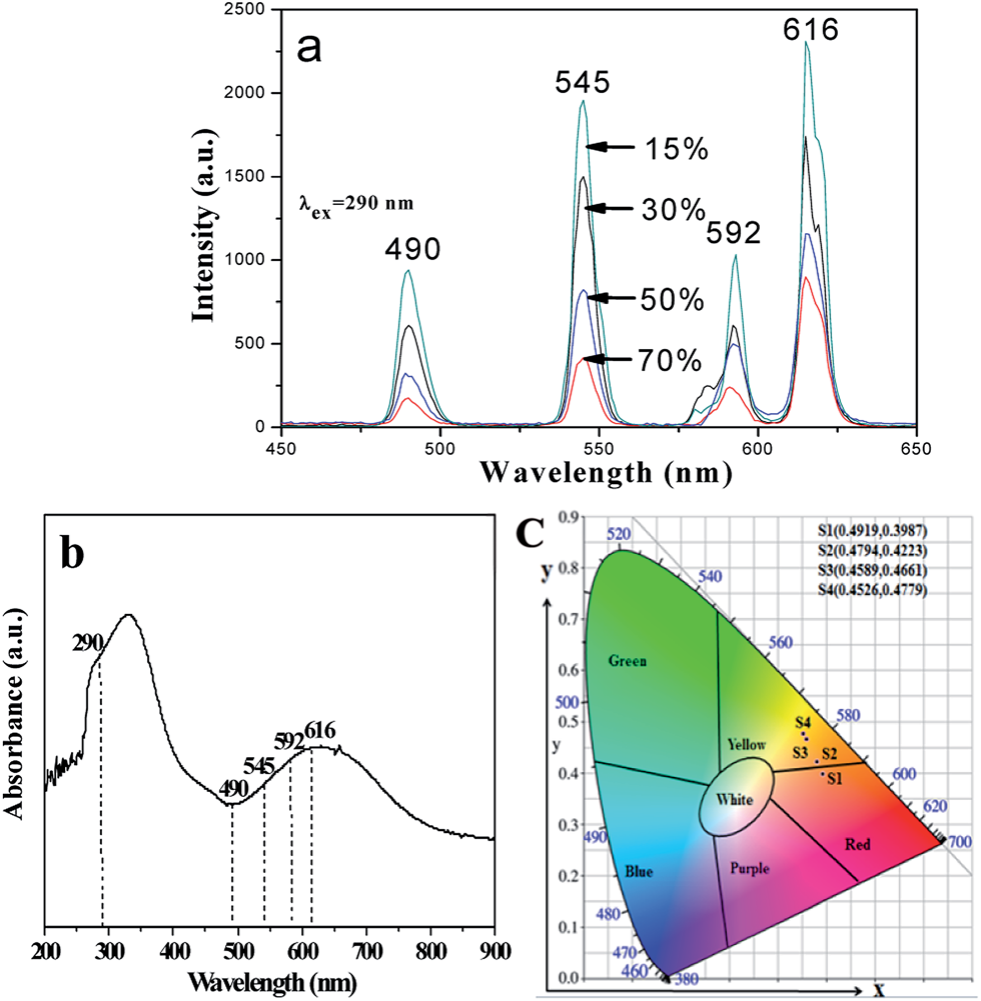
EM spectra (a) and CIE chromaticity diagram (c) of [M@Lum]//E JAM containing various mass percentages of PANI to PMMA; UV-vis absorption spectrum of PANI (b).

**Fig. 10:**
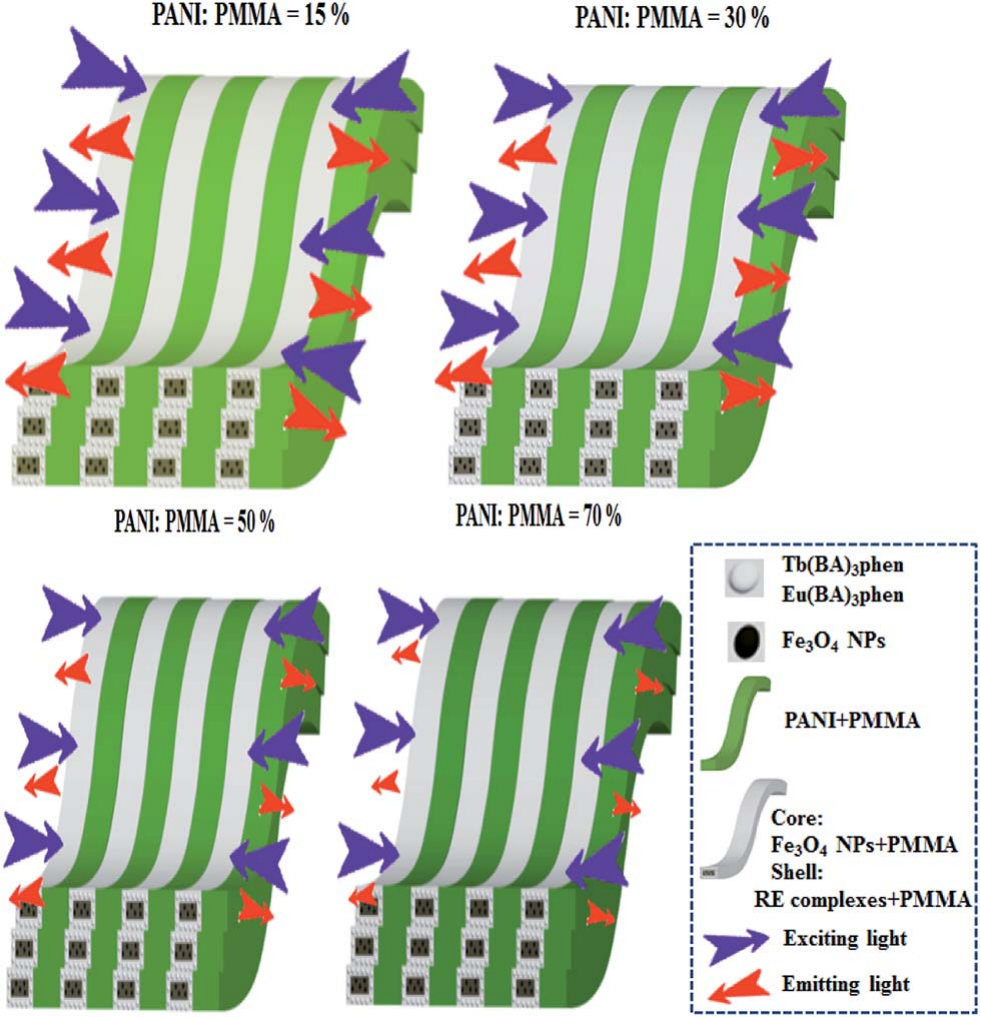
Sketch maps of the EX and EM light in [M@Lum]//E JAM containing various mass percentages of PANI to PMMA.

Meanwhile, the fluorescence properties of [M@Lum]//E JAM with various amounts of PANI (samples [S_a2_@S_b3_]//S_c*x*_, *x* = 1–4) are studied when the mass ratio of Eu(BA)_3_phen to Tb(BA)_3_phen and Fe_3_O_4_ NPs to PMMA are fixed at 1 : 1. As the PANI content increases, the EM intensities tend to decrease due to the light absorption of dark-colored PANI, as demonstrated in Fig. 9a. To illustrate the influence of different amounts of PANI on fluorescence, a schematic of EX and EM light in the array membrane with various percentages is shown in Fig. 10. As manifested in Fig. 9b, PANI strongly absorbs light in the range of 270–360 nm, 380–450 nm and 500–800 nm. Therefore, the EX and EM lights are absorbed by the PANI, resulting in reduced intensity of the EX and EM peaks. The CIE chromaticity coordinates for [M@Lum]//E JAM are shown in Fig. 9c. It is observed that with the introduction of PANI, the EM color of [M@Lum]//E JAM shifts to green, which is due to gradual intense absorption of red light by PANI.

**Fig. 11:**
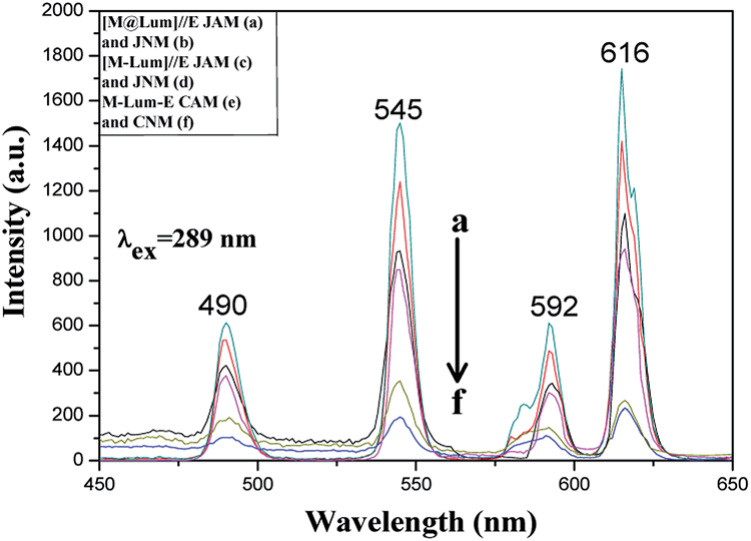
EM spectra of [M@Lum]//E JAM (a) and JNM (b), [M-Lum]//E JAM (c) and JNM (d), M-Lum-E CAM (e) and CNM (f).

**Fig. 12:**
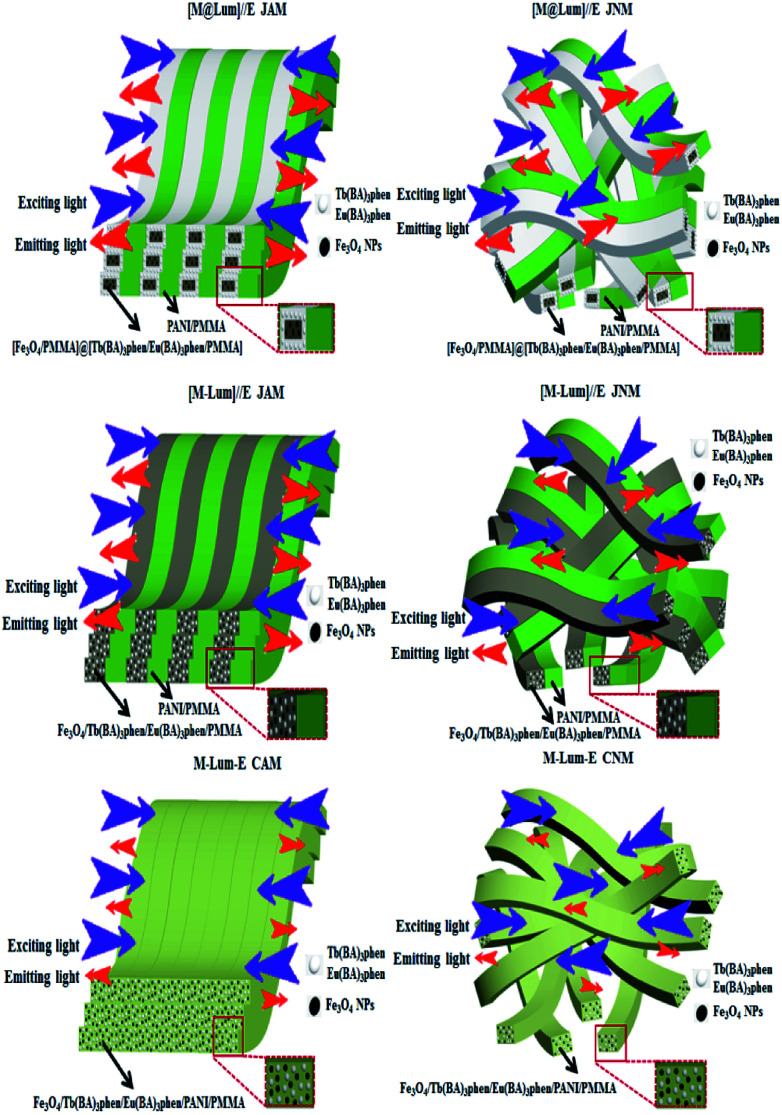
Sketch maps of EX and EM light in [M@Lum]//E JAM and contrastive samples.

Five contrastive samples were also prepared, and their fluorescence performances were in contrast with the fluorescence performance of [M@Lum]//E JAM. As seen from Fig. 11, compared with [M@Lum]//E JAM, [M@Lum]//E JNM exhibited slightly decreased fluorescence intensity. The results also indicated that other samples had weaker fluorescence intensity when they contained the same content of raw materials. Sketch maps of the EX and EM lights in [M@Lum]//E JAM and contrastive samples are shown in Fig. 12; the figure showed that the surface of [M@Lum]//E JAM was dense because it consisted of parallel Janus nanobelts. The EX light experienced difficulty while penetrating the upper layer and reached the lower Janus nanobelts; thus, the EM light was mainly emitted from the upper Janus nanobelts without reduction. As for [M@Lum]//E JNM, the disordered Janus nanobelts led to a loose membrane surface. In this situation, part of the EX light penetrated the loose upper voids to the lower Janus nanobelts. This led to the absorption of the EX light by the upper Janus nanobelts; thus, the EX light intensity weakened. In the same manner, the EX light must go through the gap in the upper layers, which is also absorbed and attenuated. In the end, the fluorescence intensity of [M@Lum]//E JNM was lower than that of [M@Lum]//E JAM. Likewise, [M-Lum]//E JAM exhibited more powerful fluorescence intensity than [M-Lum]//E JNM, and M-Lum-E CAM exhibited stronger fluorescence intensity than M-Lum-E CNM. As is seen in Fig. 11, [M@Lum]//E JAM and JNM exhibited significantly stronger luminescence intensities than other contrastive samples. As for [M@Lum]//E JAM and JNM, the constitutional unit is the coaxial nanobelt//nanobelt ([Magnetic@Luminescent]//Electrical) type Janus nanobelt, which achieves efficient isolation of PANI and Fe_3_O_4_ NPs from RE compounds, due to which the EX and EM lights absorbed by PANI and Fe_3_O_4_ NPs are significantly reduced. For [M-Lum]//E JAM and JNM, the constitutional unit is the nanobelt//nanobelt ([Magnetic-Luminescent]//Electrical) Janus nanobelt, which also benefits the isolation of RE compounds and Fe_3_O_4_ NPs from PANI; nevertheless, the RE compounds and Fe_3_O_4_ NPs are still randomly distributed together in the Fe_3_O_4_/Tb(BA)_3_phen/Eu(BA)_3_phen/PMMA nanobelts, due to which Fe_3_O_4_ NPs absorb and weaken the EX and EM lights. In terms of M-Lum-E CAM and CNM, the constitutional unit is the (Magnetic-Luminescent-Electrical) composite nanobelt, in which the RE compounds, Fe_3_O_4_ NPs and PANI are averagely dispersed as a whole in Fe_3_O_4_/Tb(BA)_3_phen/Eu(BA)_3_phen/PANI/PMMA nanobelts, with the result that the deep-colored Fe_3_O_4_ NPs and PANI strongly absorb and weaken the EX and EM lights. The above testing results demonstrate that the [M@Lum]//E Janus-structured nanobelts with three independent partitions have better performance than [M-Lum]//E Janus-structured nanobelts with two independent partitions and M-Lum-E composite nanobelts without any partition.

### Superparamagnetic property

**Fig. 13:**
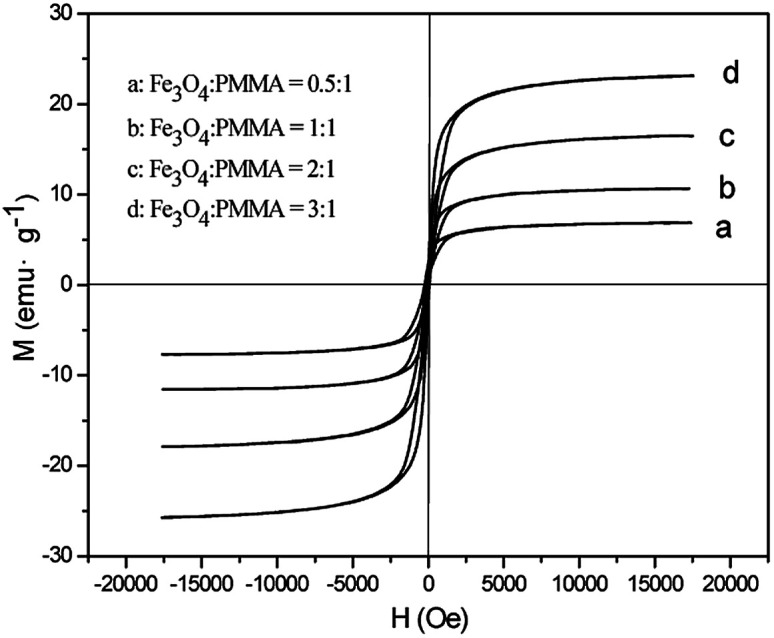
Hysteresis loops of [M@Lum]//E JAM containing various mass ratios of Fe_3_O_4_ NPs to PMMA.

Fig. 13 shows typical hysteresis loops of [M@Lum]//E JAM containing various mass ratios of Fe_3_O_4_ NPs to PMMA. As shown in Fig. 13, the samples exhibit superparamagnetic performance. The saturation magnetizations of the samples are summarized in Table 1, and the test results show that the saturation magnetization of [M@Lum]//E JAM increases as more Fe_3_O_4_ NPs are introduced into the Fe_3_O_4_/PMMA core; this indicates that the superparamagnetism of the samples can be adjusted. In addition, as seen in Table 1, the saturation magnetizations of contrastive samples and [M@Lum]//E JAM ([S_a2_@S_b3_]//S_c2_) are very close when they have similar components and constituents. Based on the above luminescence and magnetism test analyses, [M@Lum]//E JAM and contrastive samples have the same superparamagnetism, but [M@Lum]//E JAM has larger luminescence intensity than the contrastive samples, indicating that [M@Lum]//E JAM exhibits better performance.

**Table 1:** Saturation magnetizations of [M@Lum]//E JAM containing various mass ratios of Fe_3_O_4_ to PMMA and contrastive samples

Samples	Saturation magnetization (*M*_s_)/(emu g^−1^)
[S_a1_@S_b3_]//S_c2_ (Fe_3_O_4_ : PMMA = 0.5 : 1)	5.22
[S_a2_@S_b3_]//S_c2_ (Fe_3_O_4_ : PMMA = 1 : 1)	9.67
[S_a3_@S_b3_]//S_c2_ (Fe_3_O_4_ : PMMA = 2 : 1)	15.83
[S_a4_@S_b3_]//S_c2_ (Fe_3_O_4_ : PMMA = 3 : 1)	23.19
[M@Lum]//E JNM	9.71
[M-Lum]//E JAM	9.53
[M-Lum]//E JNM	9.76
M-Lum-E CAM	10.11
M-Lum-E CNM	9.34

### Electrical conduction analysis

**Fig. 14:**
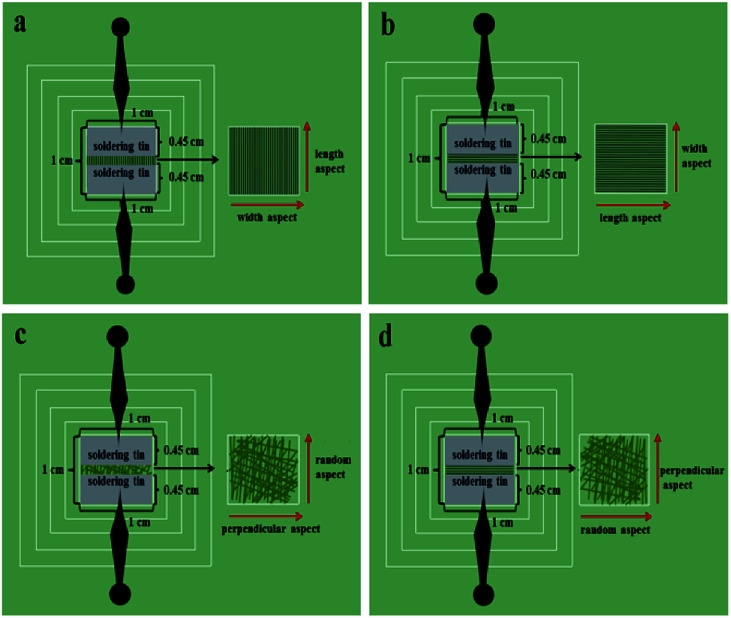
Test methods for electrical conduction along the length (a) and width (b) of the nanobelts in array membrane and along the random dimension (c) and perpendicular dimension (d) in non-array membrane.

Fig. 14 shows the test methods for the electrical conduction of array (Fig. 14a and b) and non-array membranes (Fig. 14c and d). As shown in Fig. 14, two pieces of soldering tin (0.45 cm in width and 1 cm in length) are placed on the specimen (the specimen is cut into 1 cm × 1 cm); then, the two pins of the Hall effect measurement system are connected to the two pieces of soldering tin. For the array membranes, the conductance along the length and width of the nanobelts in the membranes can be determined. For the non-array membranes, the conductance along a random dimension (equivalent to length) and the corresponding perpendicular dimension (equivalent to width) can be measured. The electrical properties of [M@Lum]//E with various amounts of PANI (samples [S_a2_@S_b3_]//S_c*x*_, *x* = 1–4) are measured, and the determined conductance values and ratios between length and width are listed in Table 2. From Table 2, it can be seen that the conductance of these array membranes can be tuned by adjusting the mass percentage of PANI to PMMA, and the conductance along the length of the [M@Lum]//E Janus nanobelts in the array membrane sharply increases from 6.24 × 10^−7^ S to 4.41 × 10^−2^ S when the mass percentage of PANI to PMMA changes from 15% to 70%. The conductance along the width dimension retains *ca.* 10 orders of magnitude, resulting in a high conductance ratio between length and width. Besides, this shows that the conductance along the length is *ca.* 8 orders of magnitude higher than that along the width when the mass percentage of PANI is fixed at 70%, which proves that [M@Lum]//E JAM has strong anisotropy in electrical conduction; as far as we know, the volume is the largest conductance ratio between the two perpendicular directions for nanobelt membrane. The results show that [M@Lum]//E JAM is electrically conducting only along the length and insulative along the width, and the Janus nanobelts have electrically conducting anisotropy that can be adjusted by varying the PANI content.

**Table 2:** Conductance volumes and anisotropy degree of [M@Lum]//E JAM

Samples	Length direction (S)	Width direction (S)	Ratio of length direction to width direction	Anisotropic degree
[S_a2_@S_b3_]//S_c1_ (PANI/PMMA: 15%)	6.24 × 10^−7^	2.48 × 10^−10^	2.52 × 10^3^	Medium
[S_a2_@S_b3_]//S_c2_ (PANI/PMMA: 30%)	8.33 × 10^−6^	1.17 × 10^−10^	7.12 × 10^4^	Medium
[S_a2_@S_b3_]//S_c3_ (PANI/PMMA: 50%)	5.75 × 10^−4^	3.24 × 10^−10^	1.77 × 10^6^	Strong
[S_a2_@S_b3_]//S_c4_ (PANI/PMMA: 70%)	4.41 × 10^−2^	1.79 × 10^−10^	2.46 × 10^8^	Very strong

**Fig. 15:**
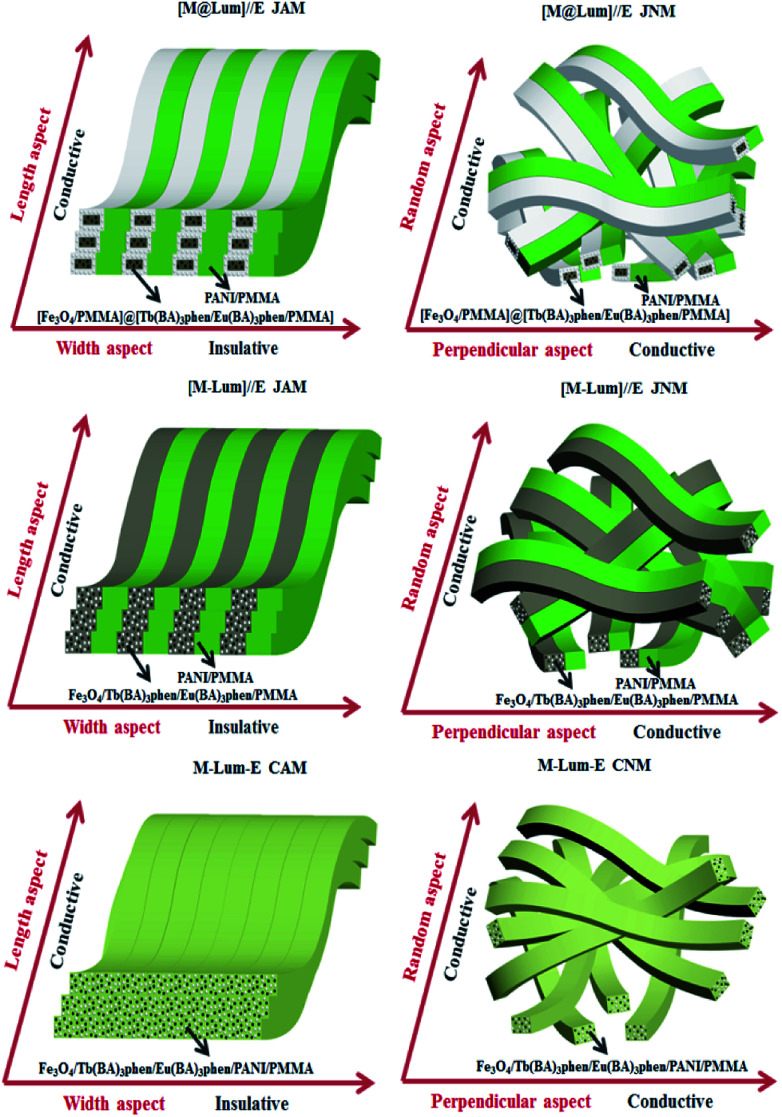
Sketch maps of electrical conduction in [M@Lum]//E JAM and contrastive samples.

Conductance and electrically conducting anisotropy of the [M@Lum]//E JAM and contrastive samples were comparatively studied when the mass percentage of PANI to PMMA was set to 30%, as seen in Table 3 and Fig. 15. In Table 3, the ratio of length to width in [M-Lum]//E JAM is 1.34 × 10^4^, which is similar to that of [M@Lum]//E JAM (7.12 × 10^4^), implying that both have strong anisotropy. In contrast, composite nanobelt array membranes only have weak electrically conducting anisotropy (5.43 × 10^1^). Fig. 15 shows the sketch maps for the electrical properties of [M@Lum]//E JAM and contrastive samples. In [M@Lum]//E JAM and [M-Lum]//E JAM, electrons can flux along the length of the Janus nanobelts without obstacles and are obstructed along the width owing to the presence of insulative [M@Lum] or [M-Lum] nanobelts, resulting in a higher conductance ratio between the two perpendicular dimensions of the two types of membranes. Compared with [M@Lum]//E JAM and [M-Lum]//E JAM, M-Lum-E CAM has significantly lower electrically conducting anisotropy. On the one hand, the electrically conducting unit is the PANI/PMMA nanobelt in [M@Lum]//E JAM and [M-Lum]//E JAM, which achieves efficient isolation of insulative RE compounds and Fe_3_O_4_ NPs from conducting PANI and guarantees the high conductance along the length of the Janus nanobelts. Nevertheless, the electrically conducting unit is Fe_3_O_4_/Tb(BA)_3_phen/Eu(BA)_3_phen/PANI/PMMA nanobelts in M-Lum-E CAM, which prevents the building of a continuous PANI conducting network and reduces the conductance of the composite nanobelts. As seen in Table 3, the conductance values along the length of [M@Lum]//E JAM and [M-Lum]//E JAM are higher than that along the length of M-Lum-E CAM. On the other hand, there are no effective insulative units among the nanobelts in M-Lum-E CAM; only the interfaces between the composite nanobelts can be used as a less effective insulating medium and thus, the conductance along the width of the nanobelts in M-Lum-E CAM is higher than that along the width in [M@Lum]//E JAM and [M-Lum]//E JAM. In brief, the insulating performance along the width of the nanobelts in M-Lum-E CAM is not strong enough. As shown in Table 3, the non-array membranes have negligible electrically conducting anisotropy. As show in Fig. 15, because the nanobelts in [M-Lum]//E JNM and M-Lum-E CNM are disordered in arrangement, the direction of current flow is disordered, which leads to electrically conducting isotropy of these non-array membranes. Under these circumstances, because electrical conduction exists in all directions of the membrane, the volume of conductance decreases in a particular dimension. From what has been discussed above, we can conclude that two key requirements must be met in the preparation of highly conducting anisotropic films. The other is that the insulator unit will be used and inserted into the insulating direction of the array film to further block the movement of electrons.

**Table 3:** Conductance volumes of [M@Lum]//E JAM and contrastive samples

Samples	Length direction (S)	Width direction (S)	Ratio of length direction to width direction	Anisotropic degree
[M@Lum]//E JAM	8.33 × 10^−6^	1.17 × 10^−10^	7.12 × 10^4^	Strong
[M@Lum]//E JNM	7.07 × 10^−7^	6.28 × 10^−7^	1.13	None
[M-Lum]//E JAM	7.42 × 10^−6^	5.51 × 10^−10^	1.34 × 10^4^	Strong
[M-Lum]//E JNM	9.02 × 10^−8^	9.67 × 10^−8^	0.93	None
M-Lum-E CAM	5.59 × 10^−7^	1.03 × 10^−8^	5.43 × 10^1^	Weak
M-Lum-E CNM	4.47 × 10^−8^	4.22 × 10^−8^	1.06	None

In combination with the above fluorescence analysis, superparamagnetism and electrically conducting anisotropy, we are pleased to discover that the fluorescence intensity of [M@Lum]//E JAM is much stronger than that of [M-Lum]//E JAM when they have similar magnetism and electrically conducting anisotropy, proving that the new [M@Lum]//E JAM have better luminescent-superparamagnetic-electrical properties than [M-Lum]//E JAM. The [M@Lum]//E Janus nanobelts with three isolated partitions are superior in structuring multifunctional nanomaterials as compared with the [M-Lum]//E Janus nanobelts with two isolated partitions and M-Lum-E composite Janus nanobelts without any partition.

### Thermal stability analysis

**Fig. 16:**
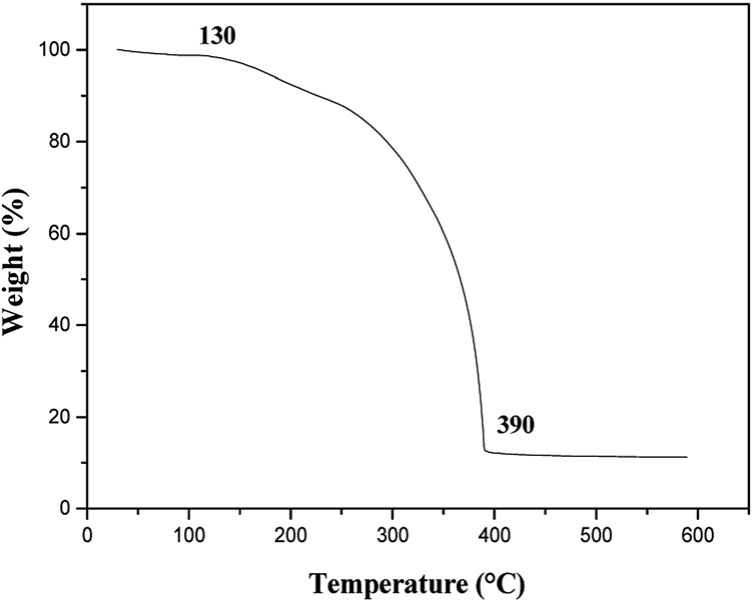
TG curve of [M@Lum]//E JAM.

The TG curve for [M@Lum]//E JAM is shown in Fig. 16. [M@Lum]//E JAM loses about 1.31% of its initial weight when the temperature is increased from room temperature to 130 °C due to volatilization of surface absorbed water. With the continuous increase in temperature, organic compounds such as Tb(BA)_3_phen, Eu(BA)_3_phen, PMMA and PANI start to decompose, and the decomposition process is completed at 390 °C. The total weight loss percentage is 87.83%. These results show that [M@Lum]//E JAM can be safely applied below 130 °C.

## Conclusions

In summary, we have established a new method to construct novel and brand-new flexible peculiar-structured [coaxial nanobelt]//[nanobelt] Janus nanobelts, which are very different from already-reported [nanobelt]//[nanobelt] structure type Janus nanobelts, *via* electrospinning technology using a newly-designed and manufactured co-axis//single-axis spinneret. With the aid of the peculiar-structured [coaxial nanobelt]//[nanobelt] Janus nanobelts, luminescent, magnetic and electrical substances are mutually isolated, and mutual interferences among them can be further reduced, due to which excellent tri-functionalities of luminescence, electricity and magnetism are concurrently integrated into the Janus nanobelts. Additionally, from the microcosmic angle, partition of only two isolated functional domains can be realized by using the already-reported common 1D Janus nanobelt; nevertheless, partition of three isolated functional domains rather than two isolated functional domains is successfully realized in the new 1D Janus nanobelt reported in this study. Therefore, the novel Janus nanobelts can also be extended to assemble various three functions to realize multi-functionalization without reducing mutual interferences. Furthermore, the peculiar-structured [coaxial nanobelt]//[nanobelt] Janus nanobelts are used to build anisotropic conductive array membranes. Because [Fe_3_O_4_/PMMA]@[Tb(BA)_3_phen/Eu(BA)_3_phen/PMMA] as insulative units are inserted into the insulative portion of the array membranes, which further blocks the movement of electrons, the largest conductivity ratio between the length and width directions of the nanobelts in the array membrane reaches up to eight orders of magnitude, which is the highest conductivity ratio between the two perpendicular dimensions for nanobelt membranes. Moreover, the electrically conducting anisotropy of Janus nanobelt array membranes can be tuned by adjusting the amount of PANI. Besides, the Janus nanobelt array membrane is simultaneously endued with tunable superparamagnetism and color-tunable fluorescence. The design philosophy and the construction technique for the novel Janus nanobelts and array membranes can afford a facile approach for the fabrication of Janus nanobelts and array membranes with other tri- or multi-functionalities.

## Conflicts of interest

There are no conflicts of interest to declare.

## Supplementary Material

RA-008-C8RA06283H-s001
