# Supplementary material for: Using special Janus nanobelt as constitutional unit to construct anisotropic conductive array membrane for concurrently affording color-tunable luminescence and superparamagnetism
Source: RSC Adv. 2018 Sep 10;8(55):31608–17. doi: 10.1039/c8ra06283h (PMC9085564; doi:10.1039/c8ra06283h)
Supplement: RA-008-C8RA06283H-s001 [file RA-008-C8RA06283H-s001.pdf]

Electronic Supplementary Material (ESI) for RSC Advances.
This journal is © The Royal Society of Chemistry 2018

Supporting Information

Utilizing Special Janus Nanobelt as Constitutional Unit to Construct Anisotropic Conductive Array Membrane Concurrently Affording Color-tunable Luminescence and Superparamagnetism

Xue Xi,[a] Wensheng Yu,[a] Qianli Ma,[a] Dan Li,[a] Xiangting Dong,*[a] Jinxian Wang,[a] Guixia Liu [a]*

[a.] Key Laboratory of Applied Chemistry and Nanotechnology at Universities of Jilin Province, Changchun University of Science and Technology, Changchun 130022 Fax: 86 0431 85383815; Tel: 86 0431 85582574; E-mail: maqianlimail@cust.edu.cn; xtdong@cust.edu.cn

Experimental Sections

Chemicals: Tb_4O_7 (99.99%), Eu_2O_3 (99.99%), benzoic acid (BA), 1,10-phenanthroline (phen), $FeCl_3·6H_2O$, $FeSO_4·7H_2O$, NH_4NO_3, polyethylene glycol (PEG, Mw ≈ 20 000), ammonia ($NH_3·H_2O$), $CHCl_3$, *N,N*-dimethylformamide (DMF), Anhydrous ethanol, methylmethacrylate (MMA), benzoylperoxide (BPO), aniline (ANI), (IS)-(+)-camphor-10 sulfonic acid (CSA), oleic acid (OA), HNO_3 and ammonium persulfate (APS) were used. All the reagents were analytically pure. Deionized water was made by ourselves.

Preparation of PMMA: PMMA was prepared in accordance with the paper [1].

Preparation of OA Modified Fe_3O_4 NPs: Fe_3O_4 NPs used in this study was prepared according to the paper [2]. The as-prepared Fe_3O_4 NPs are spherical in shape, and the mean diameter and saturation magnetization are 10 nm and 48.59 emu·g^{-1}, respectively.

Preparation of Tb(BA)$_3$phen and Eu(BA)$_3$phen Compounds: Tb(BA)$_3$phen and Eu(BA)$_3$phen powders were synthesized in the light of the reference. [3].

Preparation of Spinning Solutions for Preparing [M@Lum]//E JAM:

Table S1 Compositions and amounts of the spinning solution I

Samples	Fe_3O_4/g	$CHCl_3$/g	DMF/g	PMMA/g
S_{a1}	0.4000	9.1200	0.9026	0.8000
S_{a2}	0.8000	9.1200	0.9026	0.8000
S_{a3}	1.6000	9.1200	0.9026	0.8000
S_{a4}	2.4000	9.1200	0.9026	0.8000

Table S2 Compositions and masses of the spinning solution II

Samples	Tb(BA)$_3$phen/g	Eu(BA)$_3$phen/g	$CHCl_3$/g	DMF/g	PMMA/g
S_{b1}	0.0750	0	8.3215	0.9615	0.7500
S_{b2}	0.0525	0.0225	8.3215	0.9615	0.7500

S_{b3}	0.0375	0.0375	8.3215	0.9615	0.7500
S_{b4}	0.0225	0.0525	8.3215	0.9615	0.7500
S_{b5}	0	0.0750	8.3215	0.9615	0.7500

Table S3 Compositions and quantities of the spinning solution III

Samples	ANI/g	CSA/g	APS/g	PMMA/g	DMF/g	$CHCl_3$/g
S_{c1}	0.2100	0.2619	0.5146	1.4000	16.2000	1.8000
S_{c2}	0.4200	0.5237	1.0292	1.4000	16.2000	1.8000
S_{c3}	0.7000	0.8729	1.7153	1.4000	16.2000	1.8000
S_{c4}	0.9800	1.2220	2.4013	1.4000	16.2000	1.8000

Preparation of Spinning Solutions for Preparing Contrast Samples: The spinning solutions for preparing the [M@Lum]//E JNM were the same as those for fabricating [M@Lum]//E JAM. The spinning solutions for the M-Lum nanobelt of the [M-Lum]//E JAM and JNM were fabricated by mixing spinning solutions S_{a2} and S_{b3}, and another spinning solution for conductive nanobelt was S_{c2}. The spinning solutions for M-Lum-E CAM and CNM were prepared by blending spinning solutions S_{a2}, S_{b3} and S_{c2} together at the volume ratio of 1: 1: 1. The compositions of contrast samples were summarized in Table S4.

Electrospinning Equipments for Preparing Contrast Samples:

Home-assembled parallel spinnerets were used for preparing [M-Lum]//E JAM and JNM. Two truncated 12# stainless steel needles were bended to an angle of *ca.* 120°, and then assembled side-by-side using double-sided sticky tape to obtain parallel spinneret. The M-Lum-E CAM and CNM were fabricated by traditionally used single spinneret electrospinning setup. The electrospinning equipments, compositions of spinning solutions, spinnerets and electrospinning conditions of the contrast samples were also systematically listed in Table S4.

Table S4 Electrospinning equipments, compositions of spinning solutions, spinnerets and electrospinning conditions of samples

Samples	Electrospinning equipments	Compositions of spinning solutions	Homemade spinnerets	Electrospinning conditions
$[S_{ax}@S_{by}]//S_{cz}$ JAM (x:1-4, y:1-5, z:1-4)	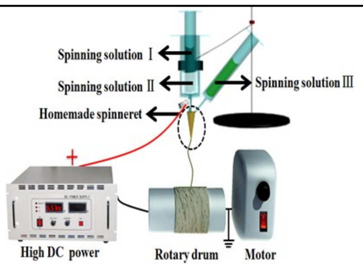	Spinning solution I: Fe_3O_4 NPs, PMMA, $CHCl_3$ and DMF<br>Spinning solution II: $Tb(BA)_3phen$, $Eu(BA)_3phen$, PMMA, $CHCl_3$ and DMF<br>Spinning solution III: CSA doped PANI, PMMA, $CHCl_3$ and DMF	Specially designed and assembled coaxis//monoaxis spinneret	Collector: aluminum rotary drum (8 cm in diameter, 20 cm in length)<br>Rotation speed: 1500 r·min^{-1}<br>Curing distance: 20 cm<br>Positive direct current voltage: 6.5 kV<br>Temperature: 22-25 °C<br>Relative humidity: 20 %-30 %
$[S_{a2}@S_{b3}]//S_{c2}$ JNM	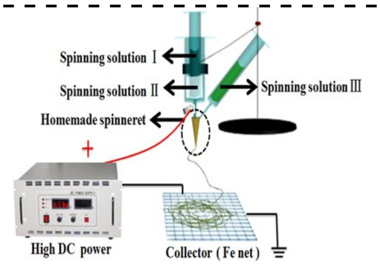	Spinning solution I: Fe_3O_4 NPs, PMMA, $CHCl_3$ and DMF<br>Spinning solution II: $Tb(BA)_3phen$, $Eu(BA)_3phen$, PMMA, $CHCl_3$ and DMF<br>Spinning solution III: CSA doped PANI, PMMA, $CHCl_3$ and DMF	Specially designed and assembled coaxis//monoaxis spinneret	Collector: flat iron net<br>Curing distance: 20 cm<br>Positive direct current voltage: 6.5 kV<br>Temperature: 22-25 °C<br>Relative humidity: 20 %-30 %
$[S_{a2}-S_{b3}]//S_{c2}$ JAM	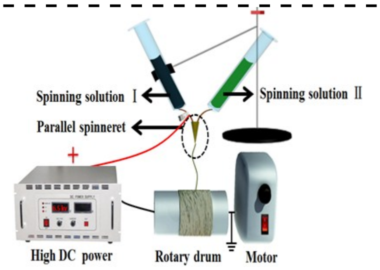	Spinning solution I: Fe_3O_4 NPs, $Tb(BA)_3phen$, $Eu(BA)_3phen$, PMMA, $CHCl_3$ and DMF<br>Spinning solution II: CSA doped PANI, PMMA, $CHCl_3$ and DMF	Parallel spinneret	Collector: aluminum rotary drum (8 cm in diameter, 20 cm in length)<br>Rotation speed : 1500 r·min^{-1}<br>Curing distance: 20 cm<br>Positive direct current voltage: 6.5 kV<br>Temperature: 22-25 °C<br>Relative humidity: 20 %-30 %
$[S_{a2}-S_{b3}]//S_{c2}$ JNM	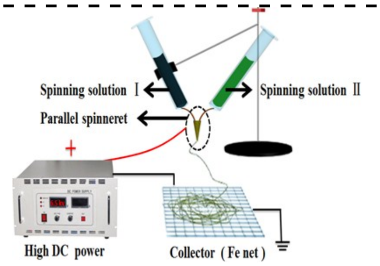	Spinning solution I: Fe_3O_4 NPs, $Tb(BA)_3phen$, $Eu(BA)_3phen$, PMMA, $CHCl_3$ and DMF<br>Spinning solution II: CSA doped PANI, PMMA, $CHCl_3$ and DMF	Parallel spinneret	Collector: flat iron net<br>Curing distance: 20 cm<br>Positive direct current voltage: 6.5 kV<br>Temperature: 22-25 °C<br>Relative humidity: 20 %-30 %
$S_{a2}-S_{b3}-S_{c2}$ CAM	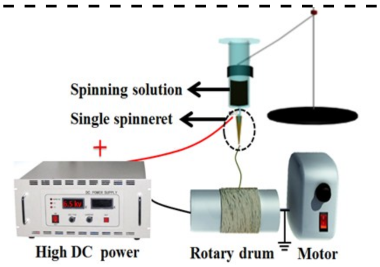	Spinning solution: Fe_3O_4 NPs, $Tb(BA)_3phen$, $Eu(BA)_3phen$, CSA doped PANI, PMMA, $CHCl_3$ and DMF	Single spinneret	Collector : aluminum rotary drum (8 cm in diameter, 20 cm in length)<br>Rotation speed : 1500 r·min^{-1}<br>Curing distance：20 cm<br>Positive direct current voltage: 6.5 kV<br>Temperature: 22-25 °C<br>Relative humidity: 20 %-30 %
$S_{a2}-S_{b3}-S_{c2}$ CNM	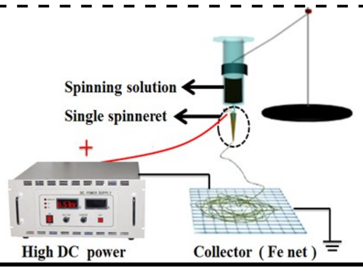	Spinning solution: Fe_3O_4 NPs, $Tb(BA)_3phen$, $Eu(BA)_3phen$, CSA doped PANI, PMMA, $CHCl_3$ and DMF	Single spinneret	Collector : flat iron net<br>Curing distance：20 cm<br>Positive direct current voltage: 6.5 kV<br>Temperature: 22-25 °C<br>Relative humidity: 20 %-30 %

Characterization Methods: The phase compositions of samples were identified by an X-ray powder diffractometer (Bruker, D8 FOCUS) with CuKα radiation, the operation voltage and current were kept at 40 kV and 20 mA, respectively. The morphologies and internal structures were observed by a field-emission scanning electron microscope (SEM, JSM-7610F) and biological microscopy (BM, CVM500E). The elemental analysis was performed by an energy dispersive spectroscope (EDS, X-MaxN80). The electrical properties were measured by a Hall effect measurement system (ECOPIA HMS-3000). The fluorescent properties were investigated by Hitachi fluorescence spectrophotometer F-7000. The UV-Vis absorption spectra were recorded by a UV-Vis spectrophotometer (SHIMADZU UV mini 1240). Then, the magnetic performances were measured by a vibrating sample magnetometer (VSM, MPMS SQUID XL). All the determinations were carried out at ambient temperature.

References

[1] J. Tian, Q.L. Ma, X.T. Dong, M. Yang, Y. Yang, J.X. Wang, W.S. Yu and G.X. Liu, J. Mater. Sci.-Mater. Eletron., 2015, **26**, 8413-8420.

[2] Y.Y. Zheng, X.B. Wang, L. Shang, C.R. Li, C. Cui, W.J. Dong, W.H. Tang and B.Y. Chen, Mater. Charact., 2010, **61**, 489-492.

[3] S. Meshkova, J. Fluoresc., 2000, **10**, 333-337.
